# PD25 Identification Of Unmet Medical Needs Through A Health Technology Assessment Process To Guide Research And Development: Experience From A University Hospital

**DOI:** 10.1017/S0266462325101608

**Published:** 2025-12-29

**Authors:** Laís Lopes, Fernanda Oliveira, Cintia Oliveira, Barbara Pacheco, Maria Angélica Ferreira

## Abstract

**Introduction:**

During the health technology assessment (HTA) process it is possible to identify unmet medical needs (UMN) that can be used to guide research and development investments. This study aimed to develop a methodology to identify and prioritize research and development themes using UMN identification in the HTA process of a teaching hospital.

**Methods:**

We reviewed and looked for UMN in all the HTA technical reports related to technology incorporation solicitations submitted between 2018 and 2023. The UMN definition used was based on that published by the US Food and Drug Administration in 2014. A prioritization process was adopted using criteria based on the World Health Organization and the Brazilian Unified Health System list of priorities for the year. The UMN were categorized by characteristics such as indication (diagnostic, therapeutic, preventive, rehabilitation) and type of diseases.

**Results:**

Thirty-seven Structured Technical Reports—formal documents submitted for evaluation of health technologies—were reviewed by two investigators. Among these, 37 UMN were identified, most of which were characterized as therapeutic needs (59.5%) or related to neurological diseases (16.2%). According to established criteria, 81.1 percent of the UMN warranted investment in research and technology development.

**Conclusions:**

The proposed methodology can reliably identify UMN by analyzing technical reports. Further improvements can make it possible to fit the process to diverse HTA systems to support research and development decisions and investments.

